# Osteogenic differentiation potential of porcine bone marrow mesenchymal stem cell subpopulations selected in different basal media

**DOI:** 10.1242/bio.053280

**Published:** 2020-10-19

**Authors:** Sangeetha Kannan, Jyotirmoy Ghosh, Sujoy K. Dhara

**Affiliations:** 1 Department of Biotechnology, Jain University, Bangalore 560011, Karnataka, India; 2 Molecular Biology Laboratory, ICAR-National Institute of Animal Nutrition and Physiology, Bangalore 560030, Karnataka, India; 3 Stem Cell Laboratory, Division of Veterinary Biotechnology, ICAR-Indian Veterinary Research Institute, Izatnagar, Uttar Pradesh 243122, India

**Keywords:** Bone marrow, Basal media, Mesenchymal stem cells, Osteogenic differentiation, Porcine

## Abstract

Multipotent porcine mesenchymal stem cells (pMSC) are invaluable for research and therapeutic use in regenerative medicine. Media used for derivation and expansion of pMSC may play an important role for the selection of MSC subpopulation at an early stage and thereby, the specific basal medium may also affect differentiation potential of these cells. The present study was undertaken to evaluate the effects of αMEM, aDMEM, M199, αMEM/M199, aDMEM/M199 and αMEM/aDMEM media on (1) porcine bone marrow MSC derivation; (2) expression of number of osteogenic markers (ALP, COL1A1, SPP1 and BGLAP) at 5th and 10th passage in pMSC before differentiation; and (3) differentiation of pMSC (at 5th passage) to osteogenic lineage. Morphological changes and matrix formation in osteogenic cells were evaluated by microscopic examination. Calcium deposits in osteocytes were confirmed by Alizarin Red S staining. Based on expression of different markers, it was evident that selection of bone marrow pMSC subpopulations was independent of basal media used. However, the differentiation of those pMSCs, specifically to osteogenic lineage, was dependent on the medium used for expansion of pMSC at the pre-differentiation stage. We demonstrated here that the pMSC grown in combined αMEM/aDMEM (1:1) medium expressed number of osteogenic markers and these pMSC underwent osteogenic differentiation most efficiently, in comparison to porcine mesenchymal stem cells grown in other media. In conclusion, osteogenic differentiation potential of pMSC maintained in αMEM/aDMEM medium was observed significantly higher compared to cells cultivated in other media and therefore, the combined medium αMEM/aDMEM (1:1) may preferentially be used for expansion of pMSC, if needed for osteogenic differentiation.

## Abstract (precis)

**Summary:** Basal media influence differentiation potential of porcine mesenchymal stem cells (pMSCs). We report here αMEM/aDMEM generates pMSC subpopulations expressing osteogenic markers and differentiate efficiently to osteogenic cells.

## INTRODUCTION

Porcine mesenchymal stem cells (pMSC) are invaluable for research and applications due to their attractive multi-lineage differentiation potential, usefulness for transgenic research (Bosch et al., 2006) and in production of cloned animals (Faast et al., 2006). Pigs, with their anatomical, genetic and pathophysiological similarities to humans, have been suggested as the best experimental model organism (Lai et al., 2002) and remain a potential cell source for the xenotransplantation studies (Comite et al., 2010). Bone marrow is the most commonly used source for MSC and the plastic adherence property of MSC is an advantage in the derivation and culture of these cells in different species. These cells are relatively easy to expand in simple culture media, and thereby provide an advantage for their applications (Girdlestone et al., 2009). For the derivation and culture of MSCs in different species, so far the following media are reported: Minimum Essential Media alpha (αMEM) (Ayatollahi et al., 2012; Ferrari et al., 2014); Dulbecco's Modified Eagle's Medium (DMEM) (Ullah et al., 2015; Sanjurjo-Rodríguez et al., 2017); DMEM/Nutrient mixture F12 (DMEM/F12) mixed media (Ullah et al., 2015); Roswell Park Memorial Institute (RPMI) 1640 medium (Ullah et al., 2015) and Tissue culture Medium M199 (M199) (Hashmani et al., 2013; Sidney et al., 2015). Equine MSC grown in DMEM/F12 and RPMI1640 were less amenable for osteogenic differentiation (Watchrarat et al., 2017). The MSC from bone marrow derived in different media show heterogeneous subpopulations, consisting of morphologically and antigenically distinct cells (Phinney and Prockop, 2007). Information on the different fractions of bone marrow MSC, their culture conditions for selective propagation and the lineage specific cell differentiation potential are not available (Szade et al., 2011).

There are 1058 registered clinical trials involved in testing the MSC-based products for the treatment of musculoskeletal tissue injuries, degenerations, diabetes, multiple sclerosis, cardiovascular diseases and liver fibrosis in human (http://clinicaltrials.gov/). Advantages of bone marrow derived MSCs are that they can be differentiated into their own lineages, the osteocytes (Jaiswal et al.,1997), adipocytes (Fink and Zachar, 2011) and chondrocytes (Solchaga et al., 2011); and trans-differentiated to myocytes (Wakitani et al., 1995), cardiomyocytes (Guo et al., 2018) and neuronal cells (Choong et al., 2007). The osteogenic differentiation potential of MSC is used for the treatment of musculoskeletal tissue injuries by direct injection at the affected site (Connolly, 1998) and as bio-scaffold-attached MSC engraft (Zhou et al., 2016). In rabbit, damaged tendon has been successfully repaired by MSC implant (Young et al., 1998). The bone marrow MSCs engraft is reported to improve the bone strength of children suffering from osteogenesis imperfecta, a group of genetic disorders in which bones of the affected patient fracture/break easily, often from mild trauma or with no apparent cause (Digirolamo et al., 1999). Osteogenesis during differentiation of MSC starts with the formation of pre-osteoblast, the common progenitor for both the osteogenic and chondrogenic cells (Nefussi et al., 1991). As the differentiation progresses, the pre-osteoblasts aggregate by a process called condensation, preceded by an increase in number of committed pre-osteoblast cells (Hall and Miyake, 1995). Further, the pre-osteoblast cells transform into osteoblasts and start secreting a non-mineralized matrix to form osteoid. Osteoid gives rise to mature osteocytes by mineralization (Franz-Odendaal et al., 2006).

The entire process is regulated by the Runt-related transcription factor 2 (RUNX2) or the core-binding factor subunit alpha-1 (CBF-alpha-1) master regulator by controlling the expression of type 1 collagen A (COL1A1), alkaline phosphatase (ALP), bone gamma carboxyglutamate (gla) protein (BGLAP)/osteocalcin and osteopontin (SPP1) genes (Kirkham and Cartmell, 2007). It is observed that the trans-activation of RUNX2 is dependent on the post-translational modification of the protein (Xiao et al., 1998) induced by bone morphogenic protein-2 (BMP-2) through p300 mediated acetylation (Jeon et al., 2006). Out of all genes regulated by RUNX2, ALP, a cell surface protein ubiquitously expressed by several cell types is used as marker for screening pre-osteoblasts (Jaiswal et al., 1997). The ALP expression gets upregulated with the progression of osteogenic differentiation of MSC within 2 days of induction (Golub and Boesze-Battaglia, 2007). The COL1A1 is an early marker of osteoblast. Increase of COL1A1 expression is observed during transformation of osteo-progenitor to pre-osteoblasts cells (Köllmer et al., 2013). The BGLAP is a small conserved non-collagenous extracellular matrix protein expressed during late osteoblast differentiation (Weinreb et al., 1990). The BGLAP/osteocalcin, expressed abundantly in the bone, is considered as the late differentiation marker and its function is associated with bone mineralization and matrix synthesis (Ducy and Karsenty, 1995). Expression of osteogenesis-related genes such as *RUNX2* and *BGLAP* is found in undifferentiated MSCs of multiple species (Ock et al., 2013), presumably due to the common mesodermal origin of MSCs. It has been observed that in porcine when osteogenesis is induced, the expression of *RUNX2* is maintained in all MSC types irrespective of tissue origin, and *BGLAP* levels increase in dermal skin-MSCs only (Wolf et al., 2016). Vacanti et al. (2005) reported that porcine MSC when expanded in advanced DMEM (aDMEM) retain multi-lineage differentiation ability in early passages whereas at late passages it loses osteo-chondrogenic differentiation ability as evident by their decrease in expression of chondrogenic marker, bone morphogenic protein (BMP-7) and osteogenic marker, ALP. Compared to DMEM, the αMEM-based pre-differentiation medium elevates the levels of osteogenic marker ALP and Collagen 1 (COL1) at passage 4 in human MSC. However, in both media groups, expression of these genes is reduced at passage 8 concomitant with the early cell detachment during osteogenic differentiation (Yang et al., 2018).

Despite their remarkable potential for treatment in varieties of diseases, the major challenge has been the difficulty in finding an appropriate *in vitro* culture system and to support their self-renewal with retention of differentiation potential in cultivated MSC. Keeping the above background in mind and the fact that basal media might play an important role in proliferation, maintenance of both undifferentiated states and differentiation potential of MSC (Brown et al., 2013), this study was designed to assess the role of each of αMEM, aDMEM, M199, αMEM/M199, aDMEM/M199 and αMEM/aDMEM media on expression of different marker genes expressed in MSC subpopulations during derivation, effects of those media on ALP, COL1A1, SPP1 and BGLAP at 5th and 10th passage of undifferentiated pMSC, and finally on outcome of osteogenic differentiation of pMSC (at 5th passage) maintained in different pre-differentiation basal media.

## RESULTS

### Expression of marker genes in pMSC

**Fig. 1.**
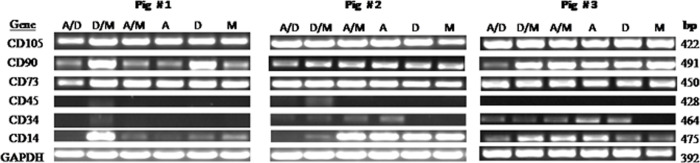
**Surface marker gene expression of porcine bone-marrow mesenchymal stem cells derived from long bones of three different animals determined by PCR amplification:** CD105 (endoglin), CD73 (SH3/4), CD90 (Thy-1), CD45 (leukocyte common antigen) CD34 (hematopoietic stem cell antigen) and CD14 (monocyte antigen) and endogenous control GAPDH (glyceraldehyde 3-phosphate dehydrogenase) genes at passage 5. Cells derived in six different basal media αMEM/aDMEM (A/D), aDMEM/M199 (D/M), αMEM/M199 (A/M), αMEM (A), advanced DMEM (D) and M199 (M) showed amplification of CD105, CD90 and CD73 in all the three animals; however, amplification of CD90 was low in M, A, A/M, and A/D for pig one, M in pig two and A/D in pig three. The leukocyte common antigen the CD45 expression was mostly not observed in all three pigs. Low level amplification of CD34 observed in D/M of pig one, in A/D, D/M, A/M and A of pig two and in A/D, D/M, A/M, A and D of pig three. Distinct CD14 expression was observed in D/M of pig one; A/D, D/M, A/M and A of pig two; and in D/M, A/M and A of pig three. In other media, CD14 amplification was either absent or low in all the three pigs.

MSC derived from all three pigs expressed CD105, CD90 and CD73 (Fig. 1). These CD molecules are considered to be positive markers for MSC. MSC, isolated from pig 1 and grown in αMEM/aDMEM, showed bands with lower intensity for CD73. Intensity of bands for CD90 also varied in cells isolated from all the three pigs and cultured across all media. Among the negative markers the general leucocytes marker CD45 expression was absent in all except in a low level in cells when cultivated in aDMEM/M199 medium. The expression of CD34 was low in cells when maintained in most of the media and no expression was observed in M199 in all the three pigs. The CD14 expression was observed in the cells derived and grown in one or multiple basal media for all the three pigs. Three different CD14^+high^, CD14^+low^ and CD14^−^ expression patterns were observed in all the three pigs (Fig. 1).

### Expression of osteogenic marker genes in pMSC

The efficiency of ALP, COL1A1, SPP1, BGLAP and GAPDH set of primers was calculated to be 2.04, 1.92, 2.09, 2.02 and 1.93, respectively. Here, expression of target genes in cells grown in different media was compared with respect to expression of the same gene in cells grown in αMEM (reference medium).

### ALP

**Fig. 2.**
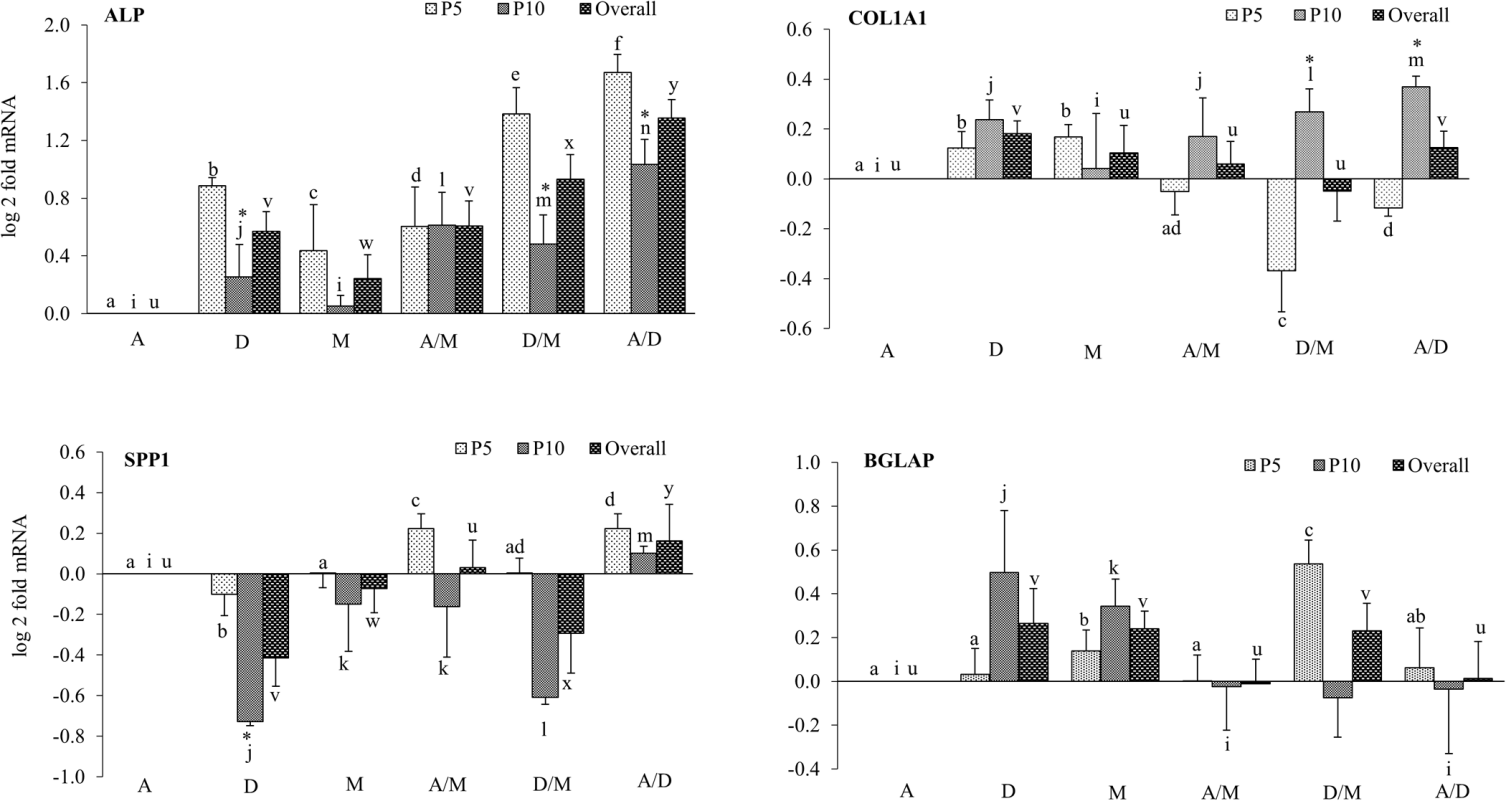
**Media and passage wise effects on osteogenesis specific gene expression in** **pMSC.** Relative mRNA expression of ALP, COL1A1, SPP1 and Bone gamma carboxyglutamate (gla) protein (BGLAP) in pMSC derived and grown in αMEM (A), aDMEM (D), M199 (M), αMEM/M199 (A/M), aDMEM/M199 (D/M), αMEM/aDMEM (A/D) media at passage 5 (P5), passage 10 (P10) and overall. The GAPDH was used as an endogenous control and the fold changes of gene expression were calculated using the method of Pfaffl (2004) considering αMEM expression as calibrator. Values under each bar are mean±s.e.m. of triplicate samples from three animals. Different letters ‘a to f’ (P5), ‘i to n’ (P10) and ‘u to z’ (overall) indicated on top of each bar represented significant difference at *P*≤0.05 between media. The ‘*’ on top of each bar represented significant difference at *P*≤0.05 between the passages.

At passage 5, expression of ALP was upregulated (*P*<0.05) in all cultures maintained with different media. Similarly, at passage 10, upregulation of ALP was also observed in all cultures except in M199. Level of expression of ALP in M199 was not different from culture maintained with αMEM medium (reference medium). Overall, a significantly higher level of ALP expression was recorded in every medium used in the current experiment (Fig. 2).

Within a single medium, a significant (indicated in Fig. 2 by an asterisk *) reduction in the level of ALP expression at passage 10 (compared to the level at passage 5) was observed in the cultures maintained in aDMEM, aDMEM/M199 and αMEM/aDMEM media. For the remaining cultures, downregulation was not significantly different (Fig. 2). The highest level of ALP expression was observed in culture maintained with αMEM/aDMEM (A/D) medium at passage 5.

### COL1A1

At passage 5, expression of COL1A1 was upregulated in two cultures (aDMEM and M199 media) and downregulated in another two cultures (aDMEM/M199 and αMEM/aDMEM media) significantly (*P*<0.05). At this passage, expression of COL1A1 in the remaining culture (αMEM/M199) was not significantly different from the level found in reference culture (αMEM). At passage 10, upregulation of COL1A1 was observed in all cultures (*P*<0.05) except M199. Overall, a significantly (*P*<0.05) higher level of COL1A1 expression was recorded in aDMEM and αMEM/aDMEM media (Fig. 2).

Within a single medium, a significantly (indicated in Fig. 2 by an asterisk *) different level of COL1A1 expression between passage 5 and passage 10 was observed only for cultures maintained in aDMEM/M199 and αMEM/aDMEM media. For the remaining cultures, difference in expression of the gene between two passage numbers was not significantly different (Fig. 2).

The highest level of upregulation and downregulation in COL1A1 expression were observed, respectively, in culture maintained with αMEM/aDMEM (A/D) medium at passage 10 and with aDMEM/M199 (D/M) medium at passage 5 (Fig. 2).

### SPP1

At passage 5, expression of SPP1 was significantly (*P*<0.05) changed in three cultures. The expression was upregulated in two cultures (αMEM/M199 and αMEM/aDMEM media) and downregulated in cells when cultivated only in aDMEM medium. At this passage, expression of SPP1 remained unchanged in the remaining two cultures when compared to reference culture (αMEM). At passage 10, except for medium αMEM/aDMEM (A/D), significant downregulation was observed in the expression SPP1 in all cultures (*P*<0.05). Overall, a significantly (*P*<0.05) lower level of SPP1 expression was recorded in four cultures viz., aDMEM, M199 and in aDMEM/M199 media; whereas, upregulation of this gene was observed only in αMEM/aDMEM culture. In the remaining αMEM/M199 culture, level of expression was not different from that of reference culture αMEM (Fig. 2).

Within a single medium, a significantly (indicated in Fig. 2 by an asterisk *) different level of SPP1 expression between passage 5 and passage 10 was observed only for two cultures: cells grown in aDMEM and in aDMEM/M199 media. For the remaining cultures, difference in expression of the gene between two passage numbers was not significantly different (Fig. 2).

The highest level of upregulation and downregulation in SPP1 expression were observed, respectively, in culture maintained in αMEM/aDMEM (A/D) medium at passage 5 and in aDMEM (D) medium at passage 10 (Fig. 2).

### BGLAP

At passage 5, expression of BGLAP was significantly (*P*<0.05) upregulated in cells propagated in M199 and aDMEM/M199 media. At this passage, expression of BGLAP remained unchanged in the remaining cultures. At passage 10, expression of the gene was upregulated only in cells cultivated with aDMEM and M199 media (*P*<0.05). Overall, a significantly (*P*<0.05) higher level of BGLAP expression was recorded in cells grown in aDMEM, M199 and in aDMEM/M199 media (Fig. 2).

Within a single medium, a significantly (indicated in Fig. 2 by an asterisk *) different level of BGLAP expression between passage 5 and passage 10 was observed only in cells grown in aDMEM/M199 medium. For the remaining cultures, difference in expression of the gene between two passage numbers was not significantly different (Fig. 2).

The highest level of upregulation of BGLAP gene was observed in culture maintained in aDMEM/M199 (D/M) medium at passage 5 (Fig. 2).

**Fig. 3.**
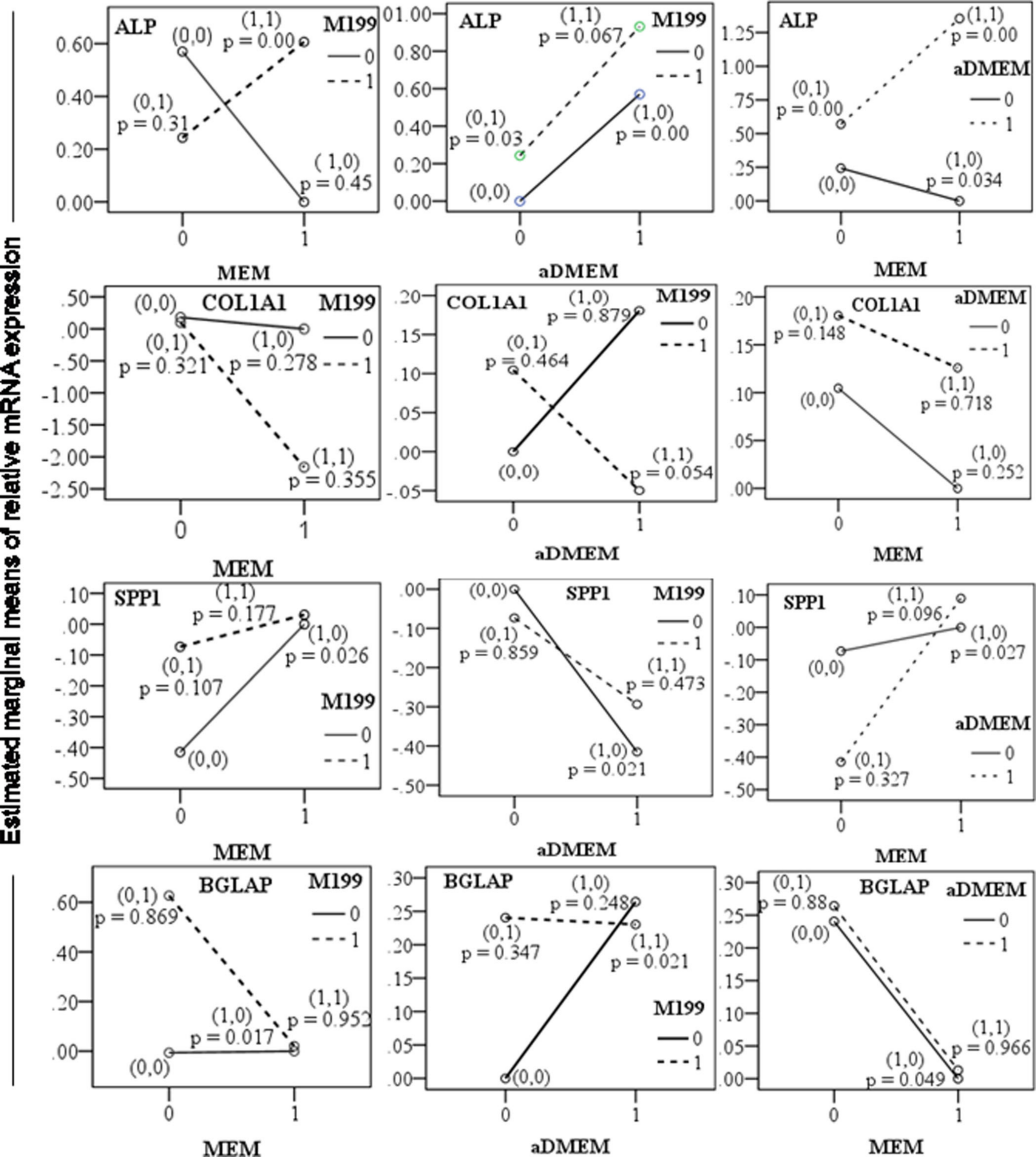
**The media interaction for the changed expression** **of ALP, COL1A1, SPP1 and BGLAP in pMSC.** The pMSC were derived and propagated in the absence (0,0), presence of single (1,0/0,1) and combinations (1,1) of two different basal media formulations. Gene expression in the absence of a particular media signified that the cells were cultured in a basal media other than the two particular media combinations. Triplicate of each sample was analyzed by established qPCR of each gene, GAPDH was used as an endogenous control gene and αMEM expression as calibrator. The *P*-values indicated in graphs were significant at *P*≤0.05. The data of log twofold mRNA expression at passage 5 and passage 10 of three pigs were pooled to obtain the marginal means for plotting the graph.

In order to understand to what extent each of the media interacted and influenced the expression of these osteogenic marker genes, we further analyzed the data. It was found that expression of early markers such as ALP and CoL1A1, interaction between two media played significant (*P*<0.05) roles, whereas for the late osteogenic markers, the effects of individual medium rather than their interaction were evident (*P*<0.05). For ALP expression, the aDMEM and all the mixed media plays supportive roles. For cells expressing COL1A1, combined aDMEM/M199 was found to be supportive. Cells expressing SPP1, a known osteogenic marker expressed in both proliferation (early) and mineralization (late) phases were supported by each of the three media. Late marker BGLAP was found to be significantly expressed in culture supported only with αMEM (Table 1 and Fig. 3). Thus, it is concluded that medium formulated by combining any two media would be supportive for proliferation but αMEM would be a better choice during differentiation.

**Table 1.**
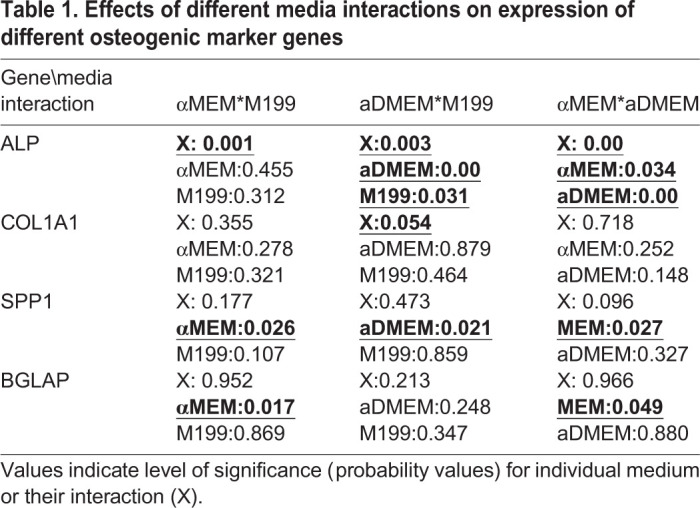
**Effects of different media interactions on expression of different osteogenic marker genes**

### Osteogenic differentiation of pMSC isolated and grown in different media

**Fig. 4.**
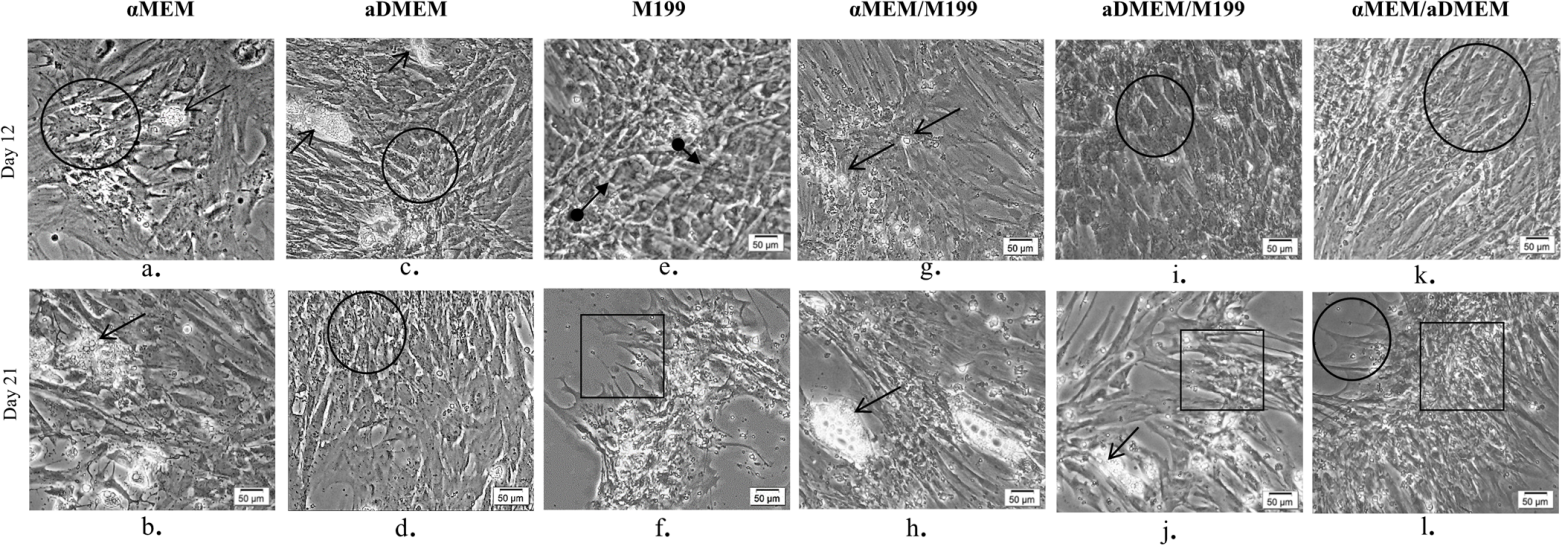
**Phase contrast images showing a comparative morphology of porcine bone marrow derived MSC on day 12 and day 21 of induction to osteogenic differentiation.** The cells from αMEM formed very few osteoblasts (circle) at day 12 (a) with also few adipogenic cells (opened arrow) as with αMEM/M199 (g) and aDMEM (c). Adipocyte-like cells were also present at day 21 in αMEM, αMEM/M199 and aDMEM/M199 (b,h,j). Cells from aDMEM, aDMEM/M199 and αMEM/aDMEM displayed changes in their morphology to cuboidal shape typical of mature osteoblasts (circle) at day 12 (c,i,k) and aDMEM and αMEM/aDMEM at day 21 (d,l). In M199, a few cells tending towards osteogenesis (closed arrow) were seen at day 12 (e) most of which matured by day 21 (f). Cells in M199, aDMEM/M199 and αMEM/aDMEM formed nodular structures due to coalescing cells with visible mineral matrix (rectangular) by day 21 of induction (f,j,l). Magnifications of respective images are as denoted by the scale bar.

**Fig. 5.**
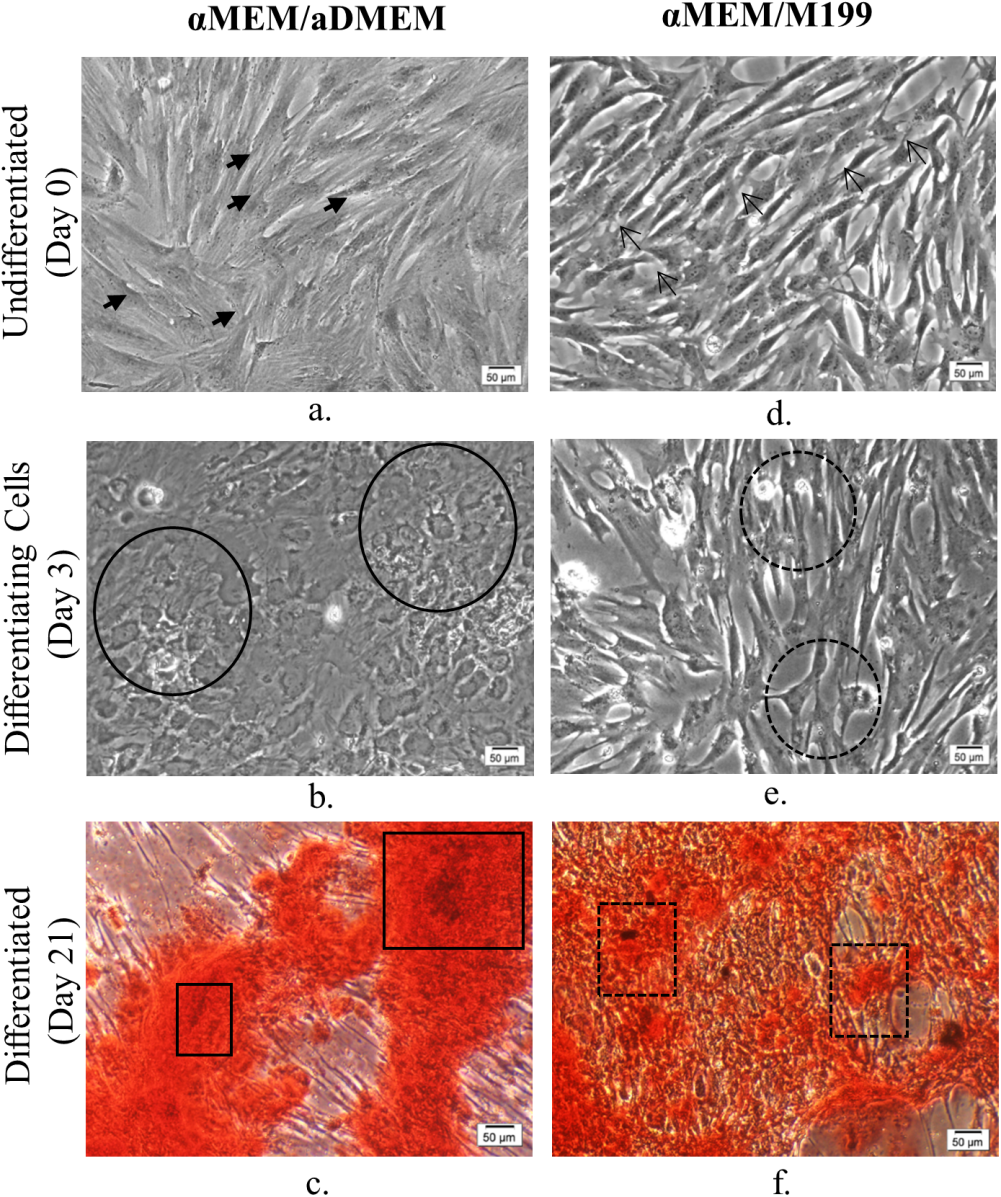
**Phase-contrast micrographs of undifferentiated (day 0), osteogenically differentiating (day 3) and differentiated (day 21) passage 5 porcine bone marrow-derived MSC.** αMEM/aDMEM grown undifferentiated cells (a) showing larger cell size (closed arrows) compared to spindle-shaped cells in αMEM/M199 (c) a typical of MSC morphology (opened arrows showing representative cells). The αMEM/aDMEM grown cells responded to osteogenic differentiation induction by changed shape to cuboidal morphology (line circles) as early as by day 3 (b) of differentiation, whereas, the cells grown in αMEM/M199 (d) did not show appreciable change in morphology (dotted circles) on the same day. The calcification assessed by Alizarin Red S stain at 21 days of osteogenic differentiation displayed heavily stained Alizarin Red S positive areas in αMEM/aDMEM grown pMSC(e) (line rectangles) whereas the pMSC grown in αMEM/M199 (f) showed patches of undifferentiated fibroblast-like cells (dotted rectangles) with very few cells displaying mineral deposition. Magnifications of respective images are as denoted by the scale bar.

During the entire 21 day period of osteogenic differentiation, pMSC isolated and grown in different media formulations underwent considerable changes in their morphology (Figs 4 and 5). Varying differentiation ability was observed in different MSC subpopulations grown in different basal media as per the set evaluation criteria. MSCs grown in αMEM had shown the whirling growth pattern of typical undifferentiated MSC by day 6. However, by day 12 few growing osteoblast cells were visible in areas of cellular aggregates and these osteoblasts attained maturity by latter half of the differentiation period. In addition, several adipocyte-like cells with small vesicles accumulation, and undifferentiated cells were also observed in the culture plate at the end of 21 days of differentiation period (Fig. 4). The pMSCs grown in aDMEM and M199 displayed changes in morphology from spindle-shaped to cuboidal by day 6 of induction and these changes became more apparent by day 12. Even by day 21 of differentiation, a number of undifferentiated cells as well as adipocyte-like cells were observed in aDMEM. Most differentiated cells in this media showed a florid array of processes, a typical of early osteoblasts phenotype. The pMSC grown in M199 medium formed the dense aggregates of mature osteoblasts in patches during differentiation. By day 21, in both aDMEM and M199 nodular structures were visible due to coalescing of cells by mineral forming matrices over the multi-layers of cells. Both cultures also contained adipocyte-like cells.

Osteoblasts were not detected in cultures with αMEM/M199 medium during the early induction period (Fig. 5). However, by day 12, a mixed population of osteoblasts, adipocyte-like cells and undifferentiated cells were seen and presence of these cells were continued until the end of day 21 of differentiation. In addition, a limited number of osteocyte-like cells were observed in the canaliculi. Interestingly, morphological changes were observed much earlier in cultures maintained with aDMEM/M199 and αMEM/aDMEM media as early as day 3 of induction (Fig. 5). The progression of differentiation was faster such that by day 12, most cells were cuboidal in shape (Fig. 4). In aDMEM/M199, a few adipocyte-like cells with small vesicles were also observed along with the osteoblast population. Although, distinct nodule aggregates exposing the bare plastic surface appeared in both the media, by day 21 a number of contaminating adipocyte-like cells were present in cultures with aDMEM/M199 medium but not in the cultures with other media. Better calcified matrix depositions were visible in αMEM/aDMEM medium with some cells transformed into stellar-shaped osteoids indicating osteoblast maturity. At pre-differentiation stage, the pMSC grown in αMEM/aDMEM medium were bigger in sizes with granular cytoplasm, an indication of skewing towards terminal differentiation (Fig. 5). During differentiation, these cells displayed a well-developed extracellular matrix and multi-layered nodular structure throughout the culture dish with no contaminating and undifferentiated cells (Fig. 4). Extensive mineralization and calcium depositions were observed in these cells after 21 days of differentiation as evidenced by the Alizarin Red S staining (Fig. 5). However, Alizarin Red S stained cells were not observed in cultures with other media up to the period of 21 days differentiation.

## DISCUSSION

The basal media might play some important roles in isolation of MSC at pre-differentiation stage and in turn, identification and use of those media should be useful for achieving lineage specific efficient differentiation to a particular cell type. We reported here that the basal media did not influence the isolation of different pMSC subpopulations, and our observations were based on expression patterns of varying marker genes in cells isolated with different media. However, the osteogenic differentiation potential of these different pMSC was dependent on the basal media used for their pre-differentiation culture. The pre-differentiation αMEM/aDMEM mixed medium was found to be the best for later osteogenic differentiation.

The larger sizes of pMSC in αMEM/aDMEM medium (Fig. 5) possibly indicated the commitment of cells towards differentiation. As a result, pMSC grown in this medium showed highest expression of ALP (Fig. 1) and also shown change in cell morphology earliest by day 3 of differentiation. These cells had retained their ability to undergo osteogenic differentiation as evident by fully differentiated osteocytes with maximum calcium depositions. At the same time, at the end of 21 days of osteogenic differentiation, the culture was devoid of undifferentiated MSC as well as contaminating adipocyte-like cells (Figs 4 and 5).The αMEM/aDMEM medium composition might have played a strong positive role for the differentiation process. The αMEM/aDMEM and αMEM media although had the similar amino acids and vitamins constituents, the cells grown in these two media did not respond similarly towards differentiation. The reason might be due to the variation in concentration of the constituents in the media (Table S1). Out of all the constituents, ascorbic acid and L-Proline present in both the media are known to influence the collagen synthesis and osteogenic differentiation differently at different concentrations (Takamizawa et al., 2004). It may be noted that in αMEM/aDMEM mixed medium, these constituents were present at 0.14 mM and 20 mg/L, exactly half the quantity of αMEM medium. This difference was likely to be responsible for the different outcome observed during osteogenic differentiation in these two media.

The ALP is an early bone differentiation marker. The mutations in ALP gene cause severe bone malformations (Liu et al., 2018). Its expression peaks highest in pre-osteoblasts formed from the osteoprogenitor cells (Jaiswal et al., 1997). The cuboidal pre-osteoblasts produce a plenty of COL1A1. As the cells progress towards more osteogenic differentiation alkaline phosphatase and osteonectin levels get elevated (Franz-Odendaal et al., 2006). Osteoblasts cells generated from pMSC grown in αMEM/aDMEM showed a larger eccentric nuclei with nucleoli, prominent rough endoplasmic reticula (ER) and Golgi areas. Such cells are expected to express a suite of bone cell markers such as bone sialoprotein, BGLAP, COL1A1 (Franz-Odendaal et al., 2006). Upon mineralization, further changes occurred such as reduced ER and Golgi areas that correspond to the decrease in protein synthesis and secretion (Franz-Odendaal et al., 2006). The inverse relationship of ALP and BGLAP expression as observed in this study has also been reported by others (Golub and Boesze-Battaglia, 2007). Cells with high ALP expression lack cell division and modulate the synthesis of BGLAP for directing differentiation of pre-osteoblasts into osteoblasts (Franz-Odendaal et al., 2006). The osteoblasts secrete active bone matrix and typically display a cuboidal morphology (Franz-Odendaal et al., 2006). The varying timing in appearance of cuboidal cells indicated different response of the pMSC towards osteogenic differentiation depending on the media used for culturing the cells. Different levels of ALP and COL1A1 expression in cells in different culture probably indicated difference in commitment of MSC towards osteogenic progression. It has been reported that increase in expression of COL1A1 results in downregulation of early osteoblast markers ALP with the increase in bone cell maturation (Malaval et al., 1994). The cells grown in αMEM/aDMEM showed the highest upregulation of ALP and had a comparable higher level of downregulation of the late osteogenic cell marker COL1A1 (Fig. 2) at passage 5 in pre-differentiation state. In addition, analysis of interaction between different media affecting the expression of target genes, revealed that αMEM/aDMEM medium influenced the ALP expression significantly (Fig. 3, Table 1). In addition, these cells responded much earlier to osteogenic differentiation indicating that they were preferentially committed towards this lineage (Fig. 5). Upregulation of COL1A1 coincided with the relatively lower expression of ALP at passage 10 in αMEM/aDMEM medium (Fig. 2) indicating existence of functional associations between these genes. Increased COL1A1 level at passage 10 in all the media (Fig. 2) might decrease the proliferation ability of the cells. Indeed decrease in cell proliferation and lower expression of different cell proliferation marker gene was observed in all the media grown pMSC at passage 10 (data under review).

BGLAP is the late differentiation marker. The siRNA mediated knockdown of osteocalcin results in the downregulation of RUNX2, ALP, osteonectin and COL1A1 indicating the role of osteocalcin is not limited to the mineralization process but also it is involved in modulating the expression of transcription factors that control osteogenic differentiation (Tsao et al., 2017). Expression of osteoblast markers such as SPP1 and BGLAP increase concomitantly with the advancement in bone formation and mineralization (Malaval et al., 1994). SPP1 is a secreted phosphoprotein whose central role is not fully characterized. In the bone matrix, osteopontin interact with integrin through its arginine-glycine-aspartate (RGD) motif and enable bone cells to adhere to the mineral matrix (Morinobu et al., 2003). Osteopontin-deficient mice showed a significant increase in the mineral depositions relative to the wild-type counterparts without altering the expression of osteogenic markers indicating its minimal role in osteoblast development (Holm et al., 2014). The role of SPP1 is suggested in osteoclastic bone resorption rather than osteoblastic bone formation (Morinobu et al., 2003). It has been reported that the expression of BGLAP requires phosphorylation of all the MAP kinases (ERK1/2, JNK1/2 and p38) whereas the SPP1 gene expression requires phosphorylation of JNK1/2 and p38 kinases (Kim et al., 2014). The readiness of cells grown in M199, aDMEM, aDMEM/M199 and αMEM/aDMEM media for undergoing osteogenic differentiation was probably associated with the upregulation of SPP1 and BGLAP genes and further corroborated with the early appearance of pre-osteoblasts cells upon induction of osteogenic differentiation (Fig. 4).

The porcine bone marrow cells derived in different media were positive for the surface markers CD105, CD90 and CD73 thus satisfied the properties of MSC according to the previous reports (Kannan et al., 2019; Dominici et al., 2006). The low level of MSC negative marker CD34 and CD45 expression as observed in our study is also reported by others (Kaiser et al., 2007; Lin et al., 2012). According to the International Society for Cellular Therapy (ISCT), although the MSC should be negative for CD14, low level expression is possible given the fact that 5% of the bone marrow MSC may have the cross reacting CD14 immuno epitopes as reported in human (Pilz et al., 2011). However, the prominent CD14 bands (Fig. 1) in pMSC subpopulation isolated from all the three animals confirm presence of highly frequent CD14+ cells in porcine bone marrow. The possibility of prominent CD14 expressing MSC can be supported by the proofs that: (1) circulating CD14 monocytes is a source of progenitors that exhibit mesenchymal cell differentiation ability (Kuwana et al., 2003); (2) the bone marrow MSC in mouse are CD14+ (Szade et al., 2011); (3) MSC derived from the peripheral blood and the adipose tissue of equine express CD14+ (Braun et al., 2010) and; (4) the CD14 expression in adipose derived equine MSC is reported to wane with the increases in passage number (McIntosh et al., 2006). Recently CD14 expression in MSCs is reported to regulate the LPS dependent TLR4 signaling pathway and dictates the differential activation through AKT, NF-kB and P38 signals (Jiang et al., 2019). However, the influence of expression of different marker genes on osteogenic differentiation of pMSCs, if any, needs to be delineated further in separate study using the pure population of cells isolated by fluorescence activated cell sorter.

Our observation indicated that none of basal media favored selection of MSC expressing a specific marker. Isolation of varying MSC subpopulation happened independently in different media just because the bone marrow harbors a heterogeneous subpopulation of morphologically and antigenically distinct cell types (Phinney and Prockop, 2007). Thus, characterization of MSC based on cell surface and other functional markers should be mandatory keeping in view that adherence of a particular subpopulation depends on their availability and chances. The MSC isolated from the same or different pigs expressing different markers could be associated with changed differentiation potential and this potential was plausibly augmented further by the media in which they were grown. It has been reported that reduced expression of CD105, CD90 or CD73 is associated with adipogenic differentiation, damage repair from bone fracture, or osteogenic differentiation through mechanical stimulation (Hung et al., 2004; Wiesmann et al., 2006). The expression of CD105 and 73 changed only in one media for one pig. However, a combined information of expression of ALP, COL1A1 as well as high and low CD90 could explain the osteogenic differentiation potential of the grown MSC in different media. The Thy-1 (CD90) is considered as a more effective biomarker for isolation of a highly osteogenic subpopulation of MSC for bone tissue engineering (Chung et al., 2013). The CD90 expression is associated with osteoprogenitor cells (Chen et al., 1999; Nakamura et al., 2010). Highest CD90 expression is reported in proliferating osteoprogenitor cells but the level decreases as the cells progress through the matrix maturation and mineralization stages (Chen et al., 1999). A decrease in CD90 expression coinciding with the increase in collagen-I and osteonectin protein expression in MSC is an indication of the presence of osteoblast-like cells (Wiesmann et al., 2006). The CD90-null mice gain weight at a faster rate, and ectopic overexpression of CD90 blocks adipogenesis (Woeller et al., 2015). A downregulation of CD90 through shRNA based knockdown favors both osteogenic and adipogenic differentiation (Moraes et al., 2016). Differentiations of MSC to osteogenic lineage might need a delicate balance of CD90 and other pre-differentiation osteogenic marker gene expression. It has been shown that CD90 is a transient marker for early differentiation and it decreases concomitantly with the increase in COL1A1 and osteonectin expression (Wiesmann et al., 2006). The activation of COL1A1 is reported to promote the osteoblastic phenotype in human osteo-progenitor cells cultured in DMEM/Hams-F12 medium (Ignatius et al., 2005). Induction of extracellular matrix (ECM) is a major activity of osteogenic cell types that occur due to the activity of COL1A1. Mutation in COL1A1 gene coding for COL1A1 protein showed reduced bone mineral density and increased osteoporotic vertebral fractures in humans (Grant et al., 1996). By blocking the interaction of integrin with COL1A1, osteocalcin and alkaline phosphatase expression was prevented (Xiao et al., 1998). The upregulation of COL1A1 in cultures with aDMEM and M199 media and downregulation of the same in cultures with aDMEM/M199 and αMEM/aDMEM media was associated, respectively, with lower and higher ALP upregulation and osteogenic differentiation potential of the cells (Fig. 2). We observed a relatively higher level of ALP expression at passage 5 compared to passage 10 contrasted the finding of Wall et al. (2007) who reported that alkaline phosphatase levels were lower between passages 5–6 as compared to passage 10. The association of high ALP expression and increased calcium deposition as reported (Wall et al., 2007) was also confirmed in our study. Vacanti et al. (2005) reported that passage 15 porcine MSC grown in aDMEM retain only the adipogenic differentiation ability not the osteogenic potential. Evidence from our study as well as from other reports indicated that the retention of differentiation potential could be the function of basal media in which cells were grown. Wall et al. (2007) reported increased osteogenic differentiation of MSCs at passage 10 when cultured in αMEM (Wall et al., 2007), whereas in a later study, it was reported that there was a loss of osteochondrogenic differentiation at passage 15 when MSC were cultured in aDMEM (Vacanti et al., 2005). The highest expression of ALP with low COL1A1 and high SPP1 in cells grown with αMEM/aDMEM mixed medium indicated that the medium might have favored better osteogenic differentiation and more calcification in the differentiated cells (Fig. 5).

Under certain favorable *in vitro* conditions, the MSC may differentiate into their own lineages (i.e. osteogenic, adipogenic and chondrogenic cells) (James, 2013) and the cells of other lineages (Wu et al., 2013). We observed here the influence of pre-differentiation media on the changes in osteogenic differentiation potential of pMSC. The best osteogenic differentiation potential in terms of earliest appearance of osteogenic cells (Fig. 5), absence of contaminating adipocytes and better calcified cells at the end of differentiation was observed in αMEM/aDMEM mixed medium supported culture (Figs 4 and 5) indicating better preparedness of these MSC toward osteogenic commitment. However, pMSC grown in some media responded with appearance of adipocyte-like cells that continued to increase (albeit not in large number) during 21 days of osteogenic differentiation. Spontaneous generation of adipocyte-like cells during osteogenic differentiation might be the proof that pMSC retain the potential to undergo adipogenic differentiation (Braun et al., 2010). However, the presence of such cells may make the differentiated cells unfit for transplantation and for therapeutic uses.

In summary, the results of this study indicated that basal media composition might not be important for selection of different MSC subpopulations from bone marrow during derivation, rather their adherence on the plastic surface as per the availability in a particular situation is due to chance. The porcine bone marrow might contain substantial CD14-expressing MSC, which needs to be studied in greater detail. Osteogenic marker gene expression and differentiation potential of pMSC varied due to the compositional differences of the basal media. The pMSC cultured in αMEM/aDMEM mixed medium is better for osteogenic differentiation.

## MATERIALS AND METHODS

All three basal media αMEM, aDMEM, M199 and osteogenic differentiation media HiOsteoXL™, used for the study were purchased from HiMedia Laboratories, Mumbai, India. The mixed media αMEM/aDMEM, αMEM/M199 and aDMEM/M199 were prepared by mixing at a 1:1 ratio. The fetal bovine serum and media supplements β-mercaptoethanol, Glutamax and antibiotic-antimycotic solution were purchased from Gibco Life Sciences, USA, and reagents from Sisco Research Laboratories (SRL), Mumbai, India.

If not stated otherwise the cells were maintained at 37°C, 5% CO_2_ and >85% relative humidity.

### Derivation and culture of MSC

The pMSC was derived from the bone marrow of the long bones of three pigs, from a local abattoir (Corporation Slaughter House, Bangalore, Karnataka, India) using a previously published protocol (Kannan et al., 2019). In brief, the bone marrow mass was collected in a sterile tube containing PBS (pH 7.2)-acid-citrate-dextrose solution and 1% antibiotic-antimycotic solution. The cells from bone marrow were mechanically dispersed and divided equally into six parts and re-suspended in αMEM, aDMEM, M199, αMEM/M199, aDMEM/M199 and αMEM/aDMEM that contained 10% FBS, 0.1% β-mercaptoethanol, 4 mM Glutamax and 1% antibiotic-antimycotic solution as supplements. The floating non-adherent cells were removed by media changes at every 48 h during derivation. The cells were passaged at 70–80% confluence by treating with 0.25% Trypsin-EDTA (#T4049, Sigma-Aldrich, USA). The cells were continuously grown until passage 10, however they were used at intermediate passages for characterization and experimentation.

### Expression of marker genes

The MSC of the three animals used for this experiment were tested for the presence or absence of CD105, CD90, CD73, CD45, CD34 and CD14 cell markers using GAPDH as endogenous control genes (Table S2) by PCR as per a previously published protocol (Kannan et al., 2019).

### Cell culture, total RNA isolation and cDNA synthesis

Passage 4 and 9 cells of each animal (*n*=3) grown in all six different media were seeded in triplicate at a density of 2×10^5^ cells/cm^2^ in sterile six-well plates and grown until 70–80% confluence with media changed every 48 h. At the termination of the culture the spent media was discarded, and adhered cells were rinsed with 1X PBS, placing the plate on a −20°C cold gel pack and treated with 0.4 ml/well TRIzol^®^ LS reagent (#10296028, Ambion™, Life Technologies, USA). The TRIzol-treated cell lysates from each well were harvested and stored at −80°C until use. Total RNA from the TRIzol-treated samples was extracted following the manufacturer's protocol. The total RNA yields and purity was determined by 260 and 280 nm absorbance using NanoDrop ND-2000 UV-Vis Spectrophotometer (Thermo Fisher Scientific, USA). The integrity of RNA in denatured samples was determined by 1% agarose gel electrophoresis in 0.2 ml 3-(N-morpholino) propane-sulfonic acid (MOPS) buffer (pH 7.0). Single-stranded cDNA from 4 µg total RNA was synthesized by reverse transcription (RT) using Revert Aid minus First Strand cDNA synthesis Kit (#K1621, Thermo Fisher Scientific) following the manufacturer's protocol.

### Quantitative PCR (qPCR) primers

The primers used were designed for the target genes ALP, COL1A1, SPP1, BGLAP and for endogenous control GAPDH (Table S3) using Primer3 web tool (http://bioinfo.ut.ee/primer3-0.4.0/primer3/) and synthesized from a local supplier (M/s Xcelris Labs limited, Ahmedabad, Gujarat, India). The amplification efficiency of all the gene primers was tested in 15 μl reaction mixtures that contained either 6.5 μl of cDNA template (50, 10, 2 and 0.4 ng total) for testing and endogenous control genes in duplicate, nuclease-free water for non-template control (NTC) in triplicate and diluted total RNA (5 ng) for negative RT (–RT) in duplicate; 0.5 μl each forward and reverse primers (5pM) and 7.5 μl SYBR 2X master mix. The amplification efficiency of each pair of gene-specific primers was calculated using the formula E=[10^(−1/slope value)^-1]×100, where slope value is slope between average cycle threshold (Ct) and log quantity.

### qPCR analysis

The amplifications of target (ALP, COL1A1, SPP1 and BGLAP) and the endogenous control (GAPDH) genes were determined in 10 μl reaction mixtures of 96 wells clear Light Cycler 480 Multi-well plate (#05102413001, Roche, USA). Triplicate 10 μl reaction mixtures were prepared that contained 4 μl either of 10 ng cDNA for gene of interest, 10 ng total RNA for –RT control and nuclease free water for NTC, 0.5 μl of each forward and reverse primers (5pM) and 5 μl of 2X FastStart SYBR Green master mix (#6924204001, Roche, USA). The PCR cycling condition set in Light Cycler 480 instrument (Roche Diagnostics, USA) was run at 95°C for 3 min initial denaturation followed by 40 cycles each comprising of 95°C denaturation for 30 s, 60°C annealing for 30 s and 72°C extensions for 30 s (image capture). The melt curve analysis was done at a initial denaturation at 95°C for 1 min followed by annealing at 55°C and increasing temperature 1°C per 10 s from 55–95°C and images captured at each 10 s intervals. The GAPDH was run for each plate and was used to normalize the variations in expression of target gene mRNA in each sample.

### Calculation of gene expression

The relative mRNA abundance of target genes in the sample was calculated from the comparative Ct using the formula (E_target_)^ΔCt_(target)_/(E_reference_)^ΔCt(_reference_) as described by Pfaffl (2004). The ΔCt_(target)_ was the difference between Ct of the target gene in calibrator (expression in αMEM) and target gene in the test (expression in other media) and ΔCt_(reference)_ is the difference between Ct of the reference gene (GAPDH) in calibrator and reference gene in the test.

### Evaluation of cell morphology during osteogenic differentiation

Passage 4 cells of different media were seeded in 1 ml of respective media at a density of 5×10^3^ cells/cm^2^ per well in a sterile 24-well plate separately for all pigs (*n*=3). The media was changed with aDMEM containing all the supplements for 48 h uniformly and allowed to be grown until about 90–95% confluence as per the subjective evaluation. The spent media was removed and replaced with equal volume of osteogenic differentiation medium in each of the wells. Fresh induction media were replaced at every 3 days until 21 days of differentiation. Changes in morphology and mineral matrix formation were recorded every day at 100x magnification using Olympus IX51 inverted microscope (Olympus, Japan). Cell images were captured at every 3 days interval using DP-73 camera and CellSens Entry 1.8 software (Olympus Soft Imaging Solutions, GmbH, Germany) and images were evaluated for the comparative differences in morphology, progression of osteogenic differentiation based on the criteria for formation of mineral matrix or nodular structures, appearance of adipocytes and the undifferentiated cells remained in culture. The images were analyzed by a single person from the sequential cell images captured at different time points.

### Confirmation of calcification in osteocytes by Alizarin Red S staining

After 21 days of differentiation, the representative cells were stained using EZStain^TM^ Osteocyte staining kit (cat. #CCK030; Himedia, Mumbai, India) to understand the deposition of mineral matrix in the differentiated cells. In brief, spent media from the wells were discarded and the cells were washed with PBS and fixed by incubating in ice-cold 70% ethanol for 1 h. The ethanol from the cell surface was washed away by rinsing with sufficient water. The staining solution (pH 4.1–4.3) from the kit was added and incubated for 45 min at room temperature in dark. Finally, the excess staining solution was removed by rinsing with distilled water. The stained cell images were captured by DP-73 camera using 200x magnifications in CellSens Entry 1.8 software (Olympus Soft Imaging Solutions, GmbH, Germany).

### Statistical analysis

The log twofold mRNA expression data of passage 5, passage 10 independently and pooled passage 5 and 10 were analyzed by full factorial design using general linear model and multivariate analysis option. The calculated log twofold gene expression was considered as dependent variables and media and passages as fixed factors. The significance of differences among the means was determined by LSD post hoc test of SPSS, 2010 Version 18.0 software. The mean±s.e.m. values were considered significant in both the analysis at *P*≤0.05.

The effects of interaction between any two media on different gene expression was analyzed considering the cells were cultured in the absence (0,0), presence of single (1,0/0,1) and combinations (1,1) of two different basal media formulations using full factorial design, gene expressions as dependent variables and media as fixed factors. Gene expression in the absence of a particular media signified that the cells were cultured in a basal media other than the two particular media used in single or in combinations. Thus, two-way ANOVA with interaction was conducted using the following model:

Y_ijk_=μ+M1_i_+M2_j_+(M1xM2)_ij_+e_ijk;_,

where Yijk represented estimated gene expression (value of log twofold); M1_i_ is the ith observation of M1 medium, M2_j_ is the jth observation of M12 medium; (M1xM2)_ij_ is the interaction between M1 and M2 at _ij_th intersection; e_ijk_ is the unexplained error of the model.

The interactions were considered significant at *P*≤0.05.

## Supplementary Material

Supplementary information
