# Supplementary material for: Osteogenic differentiation potential of porcine bone marrow mesenchymal stem cell subpopulations selected in different basal media
Source: Biol Open. 2020 Oct 19;9(10):bio053280. doi: 10.1242/bio.053280 (PMC7595700; doi:10.1242/bio.053280)
Supplement: Supplementary information [file biolopen-9-053280-s1.pdf]

Table S1: Comparison of media composition used for this study. The composition of the individual media were obtained from M/s, HiMedia Laboratories and the mixed media were calculated

Composition	Media					
	AMEM	aDMEM	M199	AMEM+M199	DMEM+M199	AMEM+DMEM
**Inorganic salts (mg/L)**						
Calcium chloride dihydrate	265	265	265	265	265	265
Ferric nitrate nonahydrate	0	0.1	0.72	0.36	0.41	0.05
Magnesium sulphate anhydrous	97.72	97.72	97.72	97.72	97.72	97.72
Potassium chloride	400	400	400	400	400	400
Sodium acetate anhydrous	0	0	50	25	25	0
Sodium bicarbonate	2200	3700	2200	2200	2950	2950
Sodium chloride	6800	6400	6800	6800	6600	6600
Sodium dihydrogen phosphate anhydrous	122	109	122	122	115.5	115.5
**Amino acids(mg/L)**						
Glycine	50	30	50	50	40	40
L-Alanine	25	0	25	25	12.5	12.5
L-Arginine hydrochloride	126	84	70	98	77	105
L-Asparagine monohydrate	50	0	0	25	0	25
L-Aspartic acid	30	0	30	30	15	15
L-Cysteine hydrochloride	100	0	0.1	50.05	0.05	50
L-Cystine dihydrochloride	31.3	62.57	26	28.65	44.285	46.935
L-Glutamic acid	75	0	67	71	33.5	37.5
L-Histidine hydrochloride monohydrate	42	42	22	32	32	42
L-Hydroxyproline	0	0	10	5	5	0
L-Isoleucine	52	105	20	36	62.5	78.5
L-Leucine	52	105	60	56	82.5	78.5
L-Lysine hydrochloride	72.5	146	70	71.25	108	109.25
L-Methionine	15	30	15	15	22.5	22.5
L-Phenylalanine	32	66	25	28.5	45.5	49
L-Proline	40	0	40	40	20	20
L-Serine	25	42	25	25	33.5	33.5
L-Threonine	48	95	30	39	62.5	71.5
L-Tryptophan	10	16	10	10	13	13
L-Tyrosine disodium salt	51.9	103.79	57.66	54.78	80.725	77.845
L-Valine	46	94	25	35.5	59.5	70
**Vitamins (mg/L)**						
L-Ascorbic acid	50	0	0.05	25.025	0.025	25
Calciferol	0	0	0.1	0.05	0.05	0
Choline chloride	1	4	0.5	0.75	2.25	2.5
D-Biotin	0.1	0	0.01	0.055	0.005	0.05
D-Ca-Pantothenate	1	4	0.01	0.505	2.005	2.5
DL-Tocopherol phosphate Disodium Salt	0	0	0.01	0.005	0.005	0

Biology Open • Supplementary information

Contd...

Composition	Media					
	AMEM	aDMEM	M199	AMEM+M199	DMEM+M199	AMEM+DMEM
Folic acid	1	4	0.01	0.505	2.005	2.5
Menadione	0	0	0.01	0.005	0.005	0
Nicotinamide	1	4	0.03	0.515	2.015	2.5
Nicotinic acid	0	0	0.03	0.015	0.015	0
Pyridoxal hydrochloride	1	4	0.03	0.515	2.015	2.5
Pyridoxine hydrochloride	0	0	0.03	0.015	0.015	0
Retinol Acetate	0	0	0.14	0.07	0.07	0
Riboflavin	0.1	0.4	0.01	0.055	0.205	0.25
Thiamine hydrochloride	1	4	0.01	0.505	2.005	2.5
i-Inositol	2	7.2	0.05	1.025	3.625	4.6
p-Amino benzoic acid (PABA)	0	0	0.05	0.025	0.025	0
Vitamin B12	1.36	0	0	0.68	0	0.68
**Others (mg/L)**						
Adenine sulphate	0	0	10	5	5	0
Adenosine monophosphate	0	0	0.2	0.1	0.1	0
Adenosine triphosphate	0	0	1	0.5	0.5	0
Cholesterol	0	0	0.2	0.1	0.1	0
Deoxyribose	0	0	0.5	0.25	0.25	0
Glucose	1000	1000	1000	1000	1000	1000
Glutathione reduced	0	0	0.05	0.025	0.025	0
Guanine hydrochloride	0	0	0.3	0.15	0.15	0
Hypoxanthine	0	0	0.354	0.177	0.177	0
Phenol red sodium salt	11	15.9	15	13	15.45	13.45
Polysorbate 80	0	0	4.9	2.45	2.45	0
Ribose	0	0	0.5	0.25	0.25	0
Thymine	0	0	0.3	0.15	0.15	0
Uracil	0	0	0.3	0.15	0.15	0
Xanthine	0	0	0.344	0.172	0.172	0
Sodium pyruvate	110	110	0	55	55	110
Lipoic acid	0.2	0	0	0.1	0	0.1
Growth Supplement mix	0	Proprietary	0	0	Proprietary	Proprietary

Biology Open • Supplementary information

Table S2: Forward (F) and reverse (R) primers used for testing the positive markers (CD105, 90 and73), negative markers (CD45, 34 and 14) and endogenous control (GAPDH) genes expression in porcine mesenchymal stem cells with their Gen Bank accession numbers, sequence, target (bp), amplicon sizes (bp) and melting temperature (Tm).

Gene	Accession no	Primers	Sequence (5' to 3')	Target (bp)	Size (bp)	Tm (°C)
CD105	NM_214031.1	F<br>R	GCTGCGCTTTGAGGGATCC<br>CCCCAGAGTCGAGATGAGATC	977 to 1398	422	61.48<br>59.11
CD73	XM_001927095.2	F<br>R	TTCTTCTCAACAGCAGCATTCC<br>CACGTGAATTCCGCCCACC	1024 to 1473	450	59.18<br>61.69
CD90	XM_005667396.1	F<br>R	GCTCTCTTGCTAACAGTCTTGC<br>CCCCAGAGTCGAGATGAGATC	146 to 550	405	59.59<br>58.11
CD45	XM_003130596.4	F<br>R	AGAATACTGGCCGTCGATGG<br>GGATCAAGATGTACTGTGCCTC	574 to 1001	428	59.61<br>58.54
CD34	XM_005656760.1	F<br>R	GACCACCGTAGCCATCTCAG<br>TCACCTCAGATTGGGCAAGG	565 to 1028	464	59.90<br>59.67
CD14	NM_001097445.2	F<br>R	ACCTTATCGACCATGGTGCG<br>TGGCCCACGACACATTACG	326 to 800	475	60.18<br>60.37
GAPDH	NM_001206359.1	F<br>R	CCATCTTCCAGGAGCGAGATC<br>AACGCAGGGATGATTTCTGGG	109 to 500	392	58.92<br>59.12

Biology Open • Supplementary information

Table S3: The list of qPCR primers used for relative quantification of osteogenic differentiation marker and endogenous control (GAPDH) genes with the accession number, sequence target (bp), amplicon sizes (bp) and melting temperature (Tm).

Genes	Accession number	Primers	Sequence (5' - 3')	Target (bp)	Size (bp)	Tm (º C)
GAPDH	NM_001206359.1	F<br>R	GGTCGGAGTGAACGGATTTG<br>AGTGGAGGTCAATGAAGGGG	108<br>218	110	58.92<br>59.00
ALP	AH012163.1	F<br>R	TCAGCTCCACCACAAACCC<br>GCGTTGGTGTTGTATGTCTTGG	411<br>500	90	59.85<br>60.35
COL1A1	XM_005668927.1	F<br>R	CCCTGCCAGATCTGTGTCTG<br>GTGGTTTCCTGGTCGGTGG	309<br>463	155	60.11<br>60.60
SPP1	NM_214023.1	F<br>R	AGCGCCACAGAATACTATTTCC<br>CAGGGCTTTCGTTGGACTTG	262<br>344	83	58.53<br>59.41
BGLAP	NM_001164004.1	F<br>R	CACCAACAGACGCACCATG<br>CTTGGACACGAAGGCTGCA	18<br>150	133	59.42<br>60.60

Biology Open • Supplementary information
